# Structural and Morphological Quantitative 3D Characterisation of Ammonium Nitrate Prills by X-Ray Computed Tomography

**DOI:** 10.3390/ma13051230

**Published:** 2020-03-09

**Authors:** Fabien Léonard, Zhen Zhang, Holger Krebs, Giovanni Bruno

**Affiliations:** 1 Bundesanstalt für Materialforschung und -prüfung, Unter den Eichen 87, 12205 Berlin, Germany; zhen.zhang@bam.de (Z.Z.); holger.krebs@bam.de (H.K.); giovanni.bruno@bam.de (G.B.); 2 Institute of Physics and Astronomy, University of Potsdam, Karl-Liebknecht-str.24-25, 14476 Potsdam, Germany

**Keywords:** ANFO, explosives, specific surface area, porosity, XCT, data processing

## Abstract

The mixture of ammonium nitrate (AN) prills and fuel oil (FO), usually referred to as ANFO, is extensively used in the mining industry as a bulk explosive. One of the major performance predictors of ANFO mixtures is the fuel oil retention, which is itself governed by the complex pore structure of the AN prills. In this study, we present how X-ray computed tomography (XCT), and the associated advanced data processing workflow, can be used to fully characterise the structure and morphology of AN prills. We show that structural parameters such as volume fraction of the different phases and morphological parameters such as specific surface area and shape factor can be reliably extracted from the XCT data, and that there is a good agreement with the measured oil retention values. Importantly, oil retention measurements (qualifying the efficiency of ANFO as explosives) correlate well with the specific surface area determined by XCT. XCT can therefore be employed non-destructively; it can accurately evaluate and characterise porosity in ammonium nitrate prills, and even predict their efficiency.

## 1. Introduction

**Figure 1:**
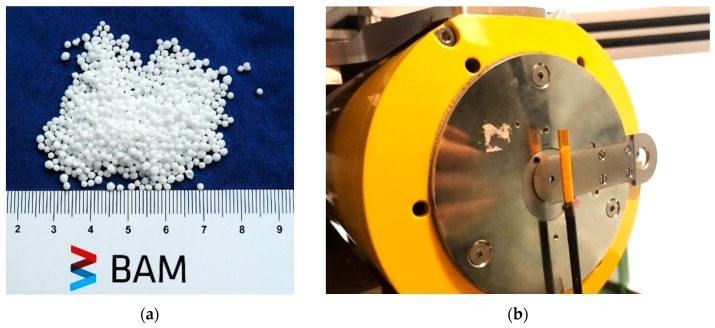
Photography of ammonium nitrate (AN) prills type E1: (**a**) loose AN prills; (**b**) AN prills paced into a polyimide tube glued onto a carbon fibre rod, prior to X-ray computed tomography (XCT) scanning.

Prilled/granulated ammonium nitrate (AN) is extensively used in the farming industry as a fertilizer and in the mining industry as an explosive. In particular, the term ANFO, for ammonium nitrate/fuel oil, specifically describes a mixture of solid ammonium nitrate prills (see Figure 1a) and diesel fuel. Thanks to its simplicity, safety, low cost, simple manufacturing process, and high blasting efficiency, ANFO represents the majority of the explosives used worldwide [1]. To quantify the importance of ammonium nitrate product usage internationally, the world’s total AN production was estimated at 15.3 million tons in 2017 [2], while the global AN market is expected to reach $6.18 billion by 2025 [3].

Ammonium nitrate prills are solid spherical pellets, ranging in diameter from tenths of millimeters to up to 2 mm, manufactured by dropping a supersaturated ammonium nitrate solution through a cooling tower 30 to 60 m in height. AN molten droplets are sprayed at the top of the tower; solidification occurs by water loss during the fall of the prill through a heated air flow. During drying, the water molecules on the surface of the AN drops evaporate, causing the initiation of crystallization on the prill surface. The water concentration gradient created between the surface and the inner part of the prill forces the internal water to migrate to the surface. The space originally occupied by the water molecules is thus replaced by air, resulting in the formation of pore channels with a complex geometry. In addition, the shrinkage in volume leads to stresses between the surface and the interior of the prill, generating cracks and flaws during solidification [4].

For the fertilizing industry, highly concentrated AN solutions (99.7% to 99.8%) are used to produce high density prills. For the mining industry, porous prills are required to promote propagation of detonation and allow a higher loading of fuel oil in ANFO mixtures: low density prills are produced for the mining industry with 96%–97% AN solutions [5]. The porosity (without packing porosity) of AN prills is approximately 20% and high quality explosive-grade prills can absorb up to about 15% fuel oil by weight [6]. The common ANFO mixture composition consists of 94.5% of AN and 5.5% of FO in weight [7]. One of the major performance predictors of the ANFO prills is the fuel oil retention, which is itself governed by the porosity of the AN prills. Presently, the oil retention capacity of ammonium nitrate in prilled and granulated forms is determined in the European Union by means of a standardised test [8]. However, this method faces technical difficulties, mainly because the porosity from different types of ammonium nitrate prills varies significantly. The porosity connected to the prill surface, i.e., open porosity, is available for oil retention. The pores not connected to the prill surface, i.e., closed porosity, are not available for oil retention but are, however, important for the explosive sensitivity to detonation [9], and the current test methods cannot account for the closed porosity.

One way to investigate and characterize the porosity of AN prills in a more accurate way is to use X-ray computed tomography (XCT). In fact, XCT allows the quantitative determination of both the open and the closed porosity, and can therefore yield a measure of the total surface area of the pore structure [10]. The main drawback of XCT is the limited resolution, when very small porosity is present. However, several strategies have been found to extract quantitative information, even in cases where single objects were not imaged [11,12]. In this paper the suitability of XCT to the non-destructive evaluation of AN prill materials is assessed. A data processing workflow is developed to extract relevant structural and morphological parameters from XCT scans of AN prills for each individual prill grain present in the XCT volume, whilst performing the segmentation on the entire 3D reconstructed volume only once. We demonstrate that differences between fertilisers and explosives and among different AN prills can be quantitatively determined from the XCT data.

## 2. Materials and Methods

### 2.1. Ammonium Nitrate Prills

Two types of AN prills were XCT scanned, the type labelled hereafter “type E” used in the mining industry as a constituent in ANFO mixtures (see Figure 1a), and the “type F”, used as a fertiliser in farming. Specimens E1 and E2 came from the same manufacturer, with similar manufacturing processes, however, E2 was expected to have higher porosity content than E1. Specimens E3 and F1 were supplied by different manufacturers than E1 and E2, and there were no available data as to their respective porosity contents. The prills, around 1.3 mm in diameter for type E and 2.4 mm in diameter for type F, were placed into a polyimide tube of 4.2 mm diameter glued onto a carbon fibre rod (see Figure 1b) and then scanned using laboratory X-ray computed tomography. All E type prills contained more than 95% pure ammonium nitrate, whilst type F contained about 40 % AN, and other potassium and ammonium salts. Typical additives were used to make the surface of the prills hydrophobic. Their production route is described in the introduction whilst further information about the products cannot be released due to confidentiality.

### 2.2. Laboratory X-Ray Computed Tomography

The AN prills were scanned on a GE V|Tome|x L 180/300 system [13] equipped with a 180 kV source, a tungsten transmission target (actual focal spot size below 2 μm as determined with JIMA test pattern RTC02), a diamond window, and a GE 2000 × 2000 pixel DXR-250 detector (GE Sensing & Inspection Technologies GmbH, Wunstorf, Deutschland). The sample projections were taken at 1800 angular positions over the 360° rotation, with increments of 0.2°, and an exposure time of 5 s per projection. The samples were placed 11.25 mm downstream from the source, with a source-detector distance of 450 mm, so that the effective magnification of 40 was obtained. The resulting pixel size was around 5.0 μm and the total scan time was 2 h 30 min. The voxel size was calibrated after the scans were performed by scanning a ball bar consisting of 2 ruby spheres glued onto a carbon fibre rod and separated by a calibrated distance of 2.273 mm ± 0.001 mm. The calibrated voxel size was determined by comparing the calibrated distance to the distance between the 2 spheres in the volumetric XCT data using VGstudio MAX version 3.2 [14]. Surface determination was performed, then 2 spheres were generated by fitting 25 points to the surface of each ruby spheres, and then the distance between the sphere centres was measured. The calculated value (5.064 µm) was then employed as voxel size for each CT scan during the data processing step. The data visualisation, processing and quantification was performed using Amira ZIB Edition version 2019.03 [15].

### 2.3. Oil Retention Tests

The oil retention tests were performed according to the Regulation (EC) 2003R2003 [8] (pp. 80–81). The measurement principle prescribes total immersion of the test sample in gas oil for a specified period of time, followed by the draining of the surplus oil under specified conditions, and finally by measuring the increase in mass of the test sample. Such tests are difficult in practice, as many parameters can influence the final increase of mass value: oil viscosity and density (which are themselves dependent on temperature), oil composition and impurity content, amount of time prills are immersed in oil, filter paper type and grade, rolling procedure for draining excess oil, etc. Since such test results are difficult to compare (e.g., between labs), it is of high importance to establish alternative techniques to quantitatively assess the explosion efficiency. We will see that XCT provides a robust quantitative assessment of the specific surface area, and of explosive efficiency.

## 3. XCT Data Processing Workflow

**Figure 2:**
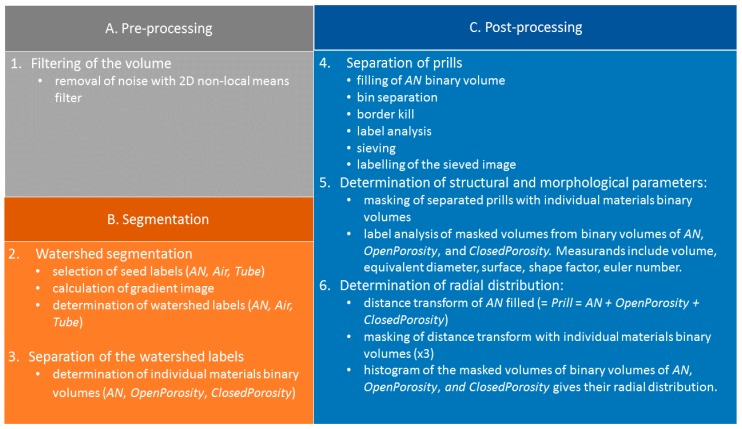
Overview of the data processing workflow.

The data processing workflow was aimed at extracting the most relevant structural parameters of the AN prills, both on a global and a local scale, and in a quantitative fashion. The entire workflow is presented in Figure 2, each step is discussed in detail below and illustrated in Video S1 (supplementary material); further details as to the Amira functions used can be found in Table A1 Appendix A.

**Figure 3:**
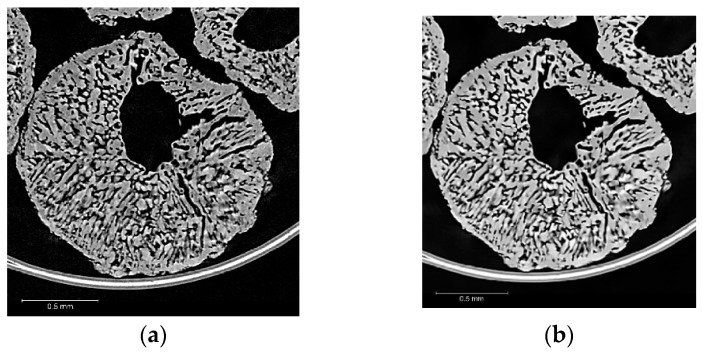

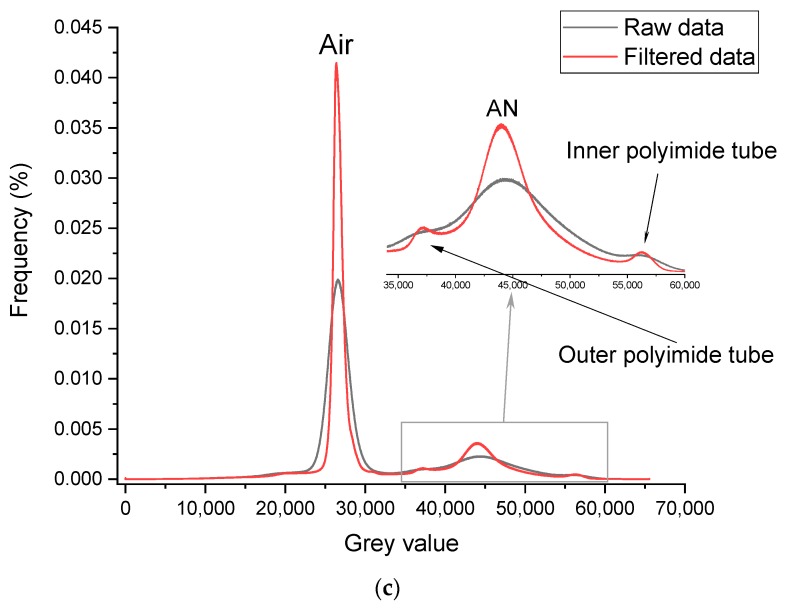
Example of 2D non-local means filtering on E2 AN prill XCT data (step 2 from Figure 2): (**a**) raw CT data; (**b**) filtered CT data; (**c**) greyscale histograms of raw and filtered XCT volumes (16 bit data).

The first step is the filtering of the data, in order to remove the noise present, whilst conserving the features of interest. For the AN prills, several filters were tested; the best filtering results were obtained with a non-local means filter (employed on all 2D slices, with a search window of 21 pixels, a local neighborhood value of 5 and a similarity value of 0.6). An example of the filtering process is presented in Figure 3: Figure 3a shows a slice of the 3D reconstruction of E2 prills, while Figure 3b represents the same slice after filtering (of the whole reconstructed volume). It can be seen on the plot (Figure 3c) that the peaks corresponding to the AN and the air appear sharper and of higher intensity after the filtering. In addition, two smaller peaks, that were previously combined into the AN peak, are now better resolved. These peaks correspond to the outer polyimide tube and the inner polyimide tube material, respectively.

**Figure 4:**
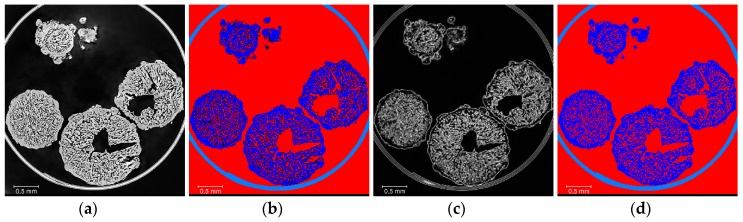
Overview of the watershed segmentation process (Figure 2 step 2): (**a**) filtered data; (**b**) label seeds (*Tube* is light blue, *AN* is dark blue, *Air* is red); (**c**) gradient image from filtered data; (**d**) labels resulting from watershed segmentation (*Tube* is light blue, *AN* is dark blue, *Air* is red), where the label seeds are taken as starting point and grown in combination with the gradient image so that all voxels in the volume get assigned to a label.

The segmentation (Figure 2, Step 2) was performed using a seed-based watershed algorithm [16], as it allows a better reproducibility between the different AN material types and lessens the impact of the threshold definition on the surface determination compared to a global threshold or ISOXX approach (see example given in Appendix B, and corresponding results in Table A2). By convention, labels and binary volumes as defined in Amira will be referred to with a name in italic, whilst a name in standard characters will be used to refer to the generic materials. Seeds of three materials were generated, namely *Tube* for the polyimide tube, *AN* for the AN prill solid material only, and *Air* for the air present in the volume, both within the prills (as closed and open porosity), and outside the prills (as air in-between the prills). An overview of the watershed segmentation process is given in Figure 4.

**Figure 5:**
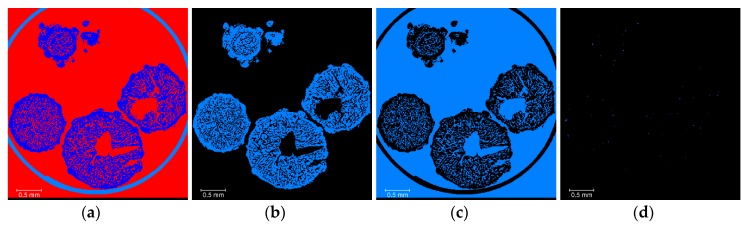
Overview of the separation of the watershed labels (Figure 2, Step 3): (**a**) labels from watershed segmentation (*Tube* is light blue, *AN* is dark blue, *Air* is red); (**b**) *AN* binary volume; (**c**) *OpenPorosity* binary volume; (**d**) *ClosedPorosity* binary volume.

Once the watershed segmentation is performed, the following individual materials binary volumes must be defined (Figure 2, Step 3): *AN*, *OpenPororisty*, and *ClosedPorosity* (as shown in Figure 5). This step is necessary for the analysis to be performed on these labels. In particular, the packing porosity (gaps between the grains) must be removed and the open and closed porosity must be separated. The *AN* binary volume is obtained directly from the watershed segmentation labels. The *ClosedPorosity* is obtained by using a *3D_fill* operation on the *AN* selection, then by subtracting the original *AN* binary volume. The *OpenPorosity* is the *Air* label minus the *ClosedPorosity* selection. Alternatively, the *OpenPorosity* selection can be obtained by selecting all the *Air* voxels connected to an *Air* voxel situated between the prills. The *ClosedPorosity* selection would then be the *Air* label minus the *OpenPorosity* selection. These two approaches are identical and yield the same results.

At this stage, it is important to bear in mind that the segmentation has been performed once on the entire volume, which means that if a label analysis was performed, it would yield a single value for each metric of interest for each binary volume (e.g., total surface area of *AN*, or total volume of *OpenPorosity* binary volumes). Although it may be correct for some quantities of some of the binary volumes, it is evidently wrong for the *OpenPorosity* binary volume, as the air in between the prills is included in the binary volume. Further processing is required to obtain the binary volumes with the correct data. To do so, the prills not fully in the 3D image must be removed, and the open porosity must be the one included within the prills, only. In addition, the data processing must be able to separate each binary volume on a prill-by-prill basis, so that the average value and standard deviation of the quantities of interest for each AN material can be determined. This is the key aspect of the work presented here, the segmentation is performed on the entire volume, then the post-processing allows extracting automatically the metrics of interest for each prill present in the 3D image, regardless of the number of prills. The workflow introduced here is an elegant alternative to a painstaking but possible (given enough time) manual segmentation and quantification of each individual prill.

**Figure 6:**
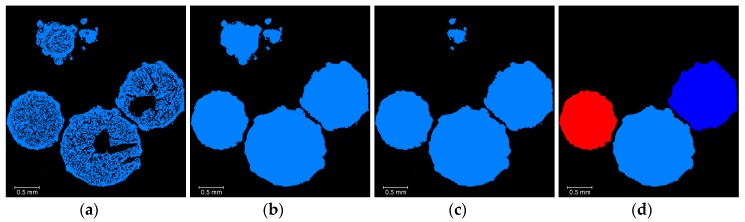
Overview of the prill separation process: (**a**) *AN* binary image; (**b**) *Prill* binary image obtained by filling operation of the AN binary image; (**c**) *Prill* binary image after a bin separation and a border kill operators; (**d**) labelled prill selection (previous *Prill* binary image after further analysis, sieving by volume to remove noise and small prill fragments, and labelling to give a volume where each prill has a unique ID).

The next step in the workflow is the separation and identification of the individual prills (Figure 2, Step 4), for which a series of six steps is necessary. First, the *AN* binary volume is selected and filled by a succession of a *3D_fill* operation, followed by three *2D_fill* operations along the three orthogonal planes of the CT volume. Then, a *Bin_Separate* operation (3D interpretation considering 26 neighbours, marker extent value of 4, repeatable algorithm) is used to separate each individual prill, followed by a *Border_kill* operation to remove prills not fully contained in the 3D image (i.e., prills that touch the borders of the 3D volume). A *Label_analysis* is performed on the resulting volume, so that each individual prill can be characterised independently. The resulting volume is sieved (*Label_sieve*) based on the volume of the prills, so that any fragment present can be removed. Finally, the prill selection obtained is labelled (*Labeling*) to give each prill a unique identifier (Figure 6d). This is required so that the material labels (*AN*, *OpenPorosity*, and *ClosedPorosity*) can be analysed on an individual prill basis and therefore some statistically relevant structural analysis performed, to highlight the differences between the AN material types.

**Figure 7:**
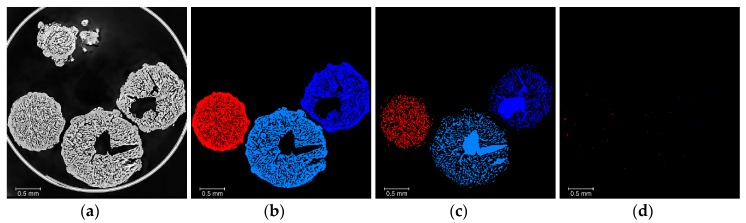
Overview of the determination of structural and morphological parameters: (**a**) filtered data; (**b**) masking of labelled prills with AN binary volume (Figure 5b and Figure 6d); (**c**) masking of labelled prills with *OpenPorosity* binary volume (Figure 5c and Figure 6d); (**d**) masking of labelled prills with *ClosedPorosity* binary volume (Figure 5d and Figure 6d).

The next step in the post-processing stage is the determination of the structural and morphological parameters from the individual materials labels (Figure 2, Step 5). Each of the individual materials binary volumes is successively masked with the labelled prill volume, as shown in Figure 7, and the following parameters are measured for each resulting labelled volume (using a *Label_analysis* operator): volume, surface area, equivalent diameter, shape factor, and Euler characteristic χ. For each parameter, the mean value is given (average of the value for each individual prill), in addition to a confidence interval corresponding to the standard deviation of the parameter.

The shape factor is defined in Equation (1),

(1) $$Shape\;Factor=\;\frac{\;Surface\;Area^{3}}{\left(36\;\times \pi \times Volume^{2}\right)}$$

where as the Euler characteristic χ (also called Euler-Poincaré characteristic or Euler number) [17] is an indicator of the connectivity of a 3D complex structure [18,19]. The Euler characteristic measures what might be called “redundant connectivity”, the degree to which parts of an object are multiply connected [20]. It is a measure of how many connections in a structure can be severed before the structure falls into two separate pieces. The Euler-Poincaré formula for a 3D object X is given in Equation (2):

(2) $$\chi \left(X\right)=\beta_{0}-\beta_{1}+\beta_{2}$$

where $\beta_{0}$ is the number of objects (the number of connected components), $\beta_{1}$ the connectivity, and $\beta_{2}$ the number of enclosed cavities. The Euler characteristic is used here as an indicator of the complexity of the topology of the AN prill and associated open porosity. The specific surface value was calculated for each prill from the ratio between the surface area of the *AN* (strictly speaking, the surface area of *AN* minus the surface area of *ClosedPorosity* as only open porosity is here of interest, since it provides the surface available for reaction with the oil) and the AN material volume, hence the unit in mm^2^/mm^3^ (unit of surface area per unit of volume). It is here relevant to mention that it is not possible to convert this into a more common mm^2^/g (unit of surface area per unit of mass), as the density of the bulk material is not well known and cannot be accurately defined from the XCT data for each individual prill.

**Figure 8:**
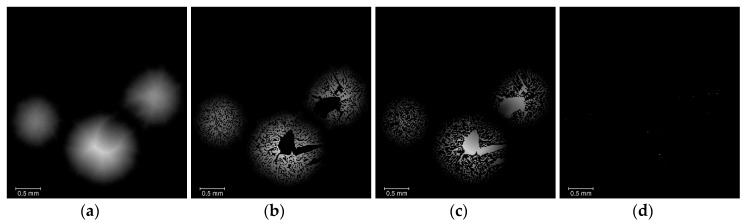
Overview of the determination of radial distribution: (**a**) distance transform of prills; (**b**) masking of prill distance transform with AN binary volume; (**c**) masking of prill distance transform with *OpenPorosity* binary volume; (**d**) masking of prill distance transform with *ClosedPorosity* binary volume. The radial distributions are obtained by plotting the histogram of the respective volumes.

The final post-processing step is dedicated to obtaining the radial distribution of the different phases within the prills (Figure 2 step 6). This is useful as it can help distinguishing between two prill microstructures that could have an overall similar open porosity content, whilst having the pore network radially distributed in a different manner. As shown in Figure 8, a *Distance_transform* operation (Euclid type) is used on the *Prill* selection, then the output volume is masked with the respective *AN*, *OpenPorosity*, and *ClosedPorosity* binary volumes. The distance transform measures the distance of each object point from the nearest boundary [21,22]. The radial distributions (i.e., radial volume fractions) are given by the histograms (voxel count versus distance) of the corresponding masked volumes. In this case however, all the prills present in the volume are processed at the same time, thus only a mean value of the radial distributions averaged over all the prills can be obtained. It is not possible with the actual data processing software to perform the distance transform onto the labelled AN prills: the information contained in the voxels of the 3D image are either the distance to the nearest voxel selected as background (i.e., distance transform from the respective binary volumes) or a label number (i.e., labelled prill volume); these two data fields are mutually exclusive and cannot be combined. As the grain sizes of the different types of AN prills (from F1 through E3) differ slightly, the distances to the outer surface of the prills were normalized for easier comparison between the different materials.

## 4. Experimental Results and Quantitative Analysis

**Figure 9:**
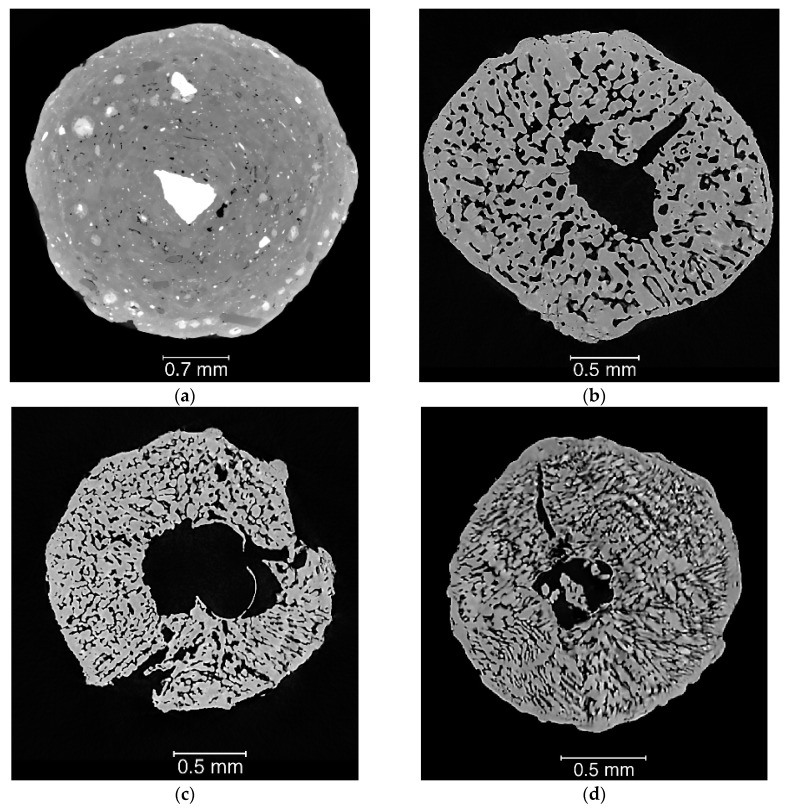
Example of CT slice taken in the middle plane of a single prill for each specimen under investigation: (**a**) specimen F1 (scale bar is different from other samples); (**b**) specimen E1; (**c**) specimen E2; (**d**) specimen E3.

As shown in Figure 9, representative 2D slices taken in the middle plane of each specimen give a good qualitative overview of the structure of the different types of AN prills. For all type E samples, there is a large cavity close to the centre of the prill, with finer pores located throughout the remainder of the prill. E1 has a pore network with more rounded edges, whilst E2 and E3 have a more dendritic-like pore structure expanding radially. Sample F1 displays a totally different structure, with several large (500 µm to 1 mm) high density inclusions and few individual pores dispersed throughout the prill. Even though the porosity can be seen from a 2D XCT slice, similarly to a SEM image, it is not possible to determine if the visible porosity is open (connected to the exterior and therefore susceptible to interact with oil) or closed (no reaction with oil possible but influence on detonation susceptibility). This is a prime example to showcase the need for 3D visualisation and associated qualitative/quantitative measurements.

**Figure 10:**
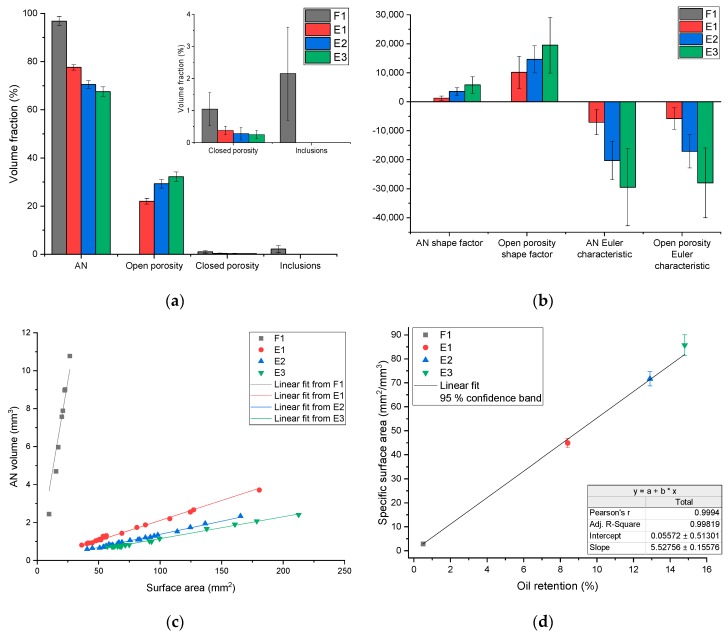
Structural and morphological results: (**a**) volume fraction; (**b**) shape factor and Euler characteristic χ for *AN* and *OpenPorosity* (no *OpenPorosity* parameters calculated for F1 as the open porosity is too small to be significant from a morphological point of view, F1 AN shape factor is 1.25 and F1 Euler characteristic is −5.88); (**c**) correlation between *AN* volume and surface area; (**d**) correlation between specific surface area and oil retention.

The structural and morphological analysis (Figure 2, Step 6) provides measurements of the volume fraction, surface area, shape factor, Euler characteristic, and specific surface area. An overview of the results is gathered in Figure 10. In terms of volume fraction (Figure 10a), the fertiliser sample (sample F1) is the sample with the highest volume fraction of AN (96.8%) and the lowest volume fraction of open porosity (below 0.02% ± 0.01%). It is the only sample displaying high density inclusions (2.2%) and has the highest volume fraction of closed porosity (1.0%), with a value more than twice higher than that of the other materials. The AN used for explosives (samples E1 through E3) have significantly lower volume fractions of AN, with values decreasing for sample E1 to E3 (see Figure 10a). Conversely, the volume fractions of open porosity are much higher, to allow the retention of the fuel oil: the open porosity increases from E1 to E3. Regarding the closed porosity, the volume fractions for all E samples are well below 1%.

For the morphological analysis (Figure 10b), either the AN or the open porosity can be selected. The analysis of the AN yields more representative results, but it is also interesting to consider the open porosity itself, as it is a true representation of the volume available for the fuel oil to soak into the AN prill. The AN shape factor values increase from F1 to E3 materials (see Figure 10b). The value for F1 is low (1.25), indicating that the prill has a shape close to that of a sphere. For the explosive materials, the values increase to very large values, indicating a dramatic increase in complexity of the AN prill shape. The increase in shape complexity from sample E1 through to E3 is further demonstrated by the open porosity shape factors, which have a similar trend and higher values. Another way to describe the morphology of the AN prills is to consider the Euler characteristic χ. For both the AN and the open porosity, the Euler characteristic values decrease from sample F1 to sample E3, with both values being similar. This evolution is consistent with an increase in complexity of the open porosity network, in good agreement with the open porosity contents and shape factors measured.

Figure 10c shows the correlation between *AN* volume and surface area for each prill of the XCT volume. It is clear that the F1 material has a much greater AN volume than the explosive materials, but with an associated low surface area. The explosive materials have greater surface area (35 mm^2^ up to 210 mm^2^), but a lower AN volume, due to the lower prill size. As the porosity contents vary somewhat significantly among the explosive materials (10% difference between E1 and E3), whilst the grain size remains comparable, a better metric to compare the materials is the specific surface, defined as the surface area of *AN* per unit of *AN* volume. Figure 10d shows the linear relationship between the specific surface values determined by X-ray computed tomography and the oil retention values determined according to the European regulation [8]. With this result, we demonstrate that XCT could be used to predict the performance of explosives over a very wide range of porosity content.

**Figure 11:**
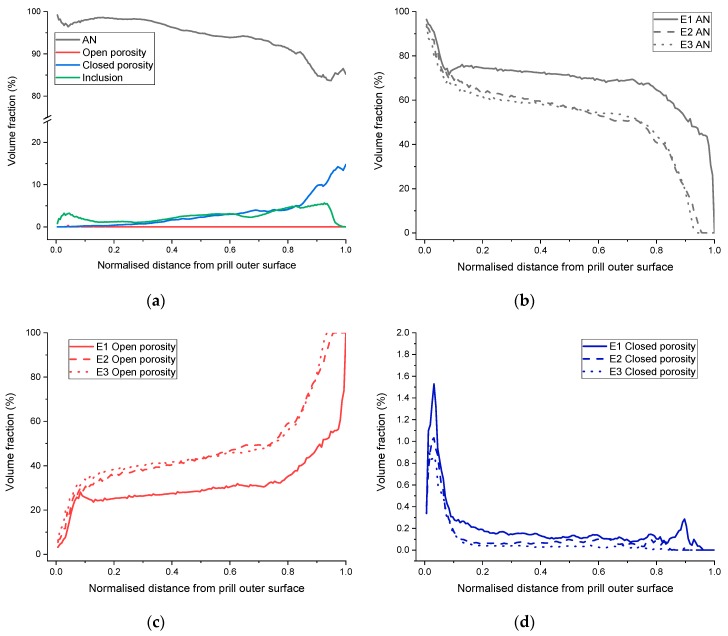
Plots from radial distribution: (**a**) specimen F1; (**b**) AN for type E specimens; (**c**) open porosity for type E specimens; (**d**) Closed porosity for type E specimens.

Based on a distance transform operator, the radial volume fraction of each phase can be determined (Figure 2 step 6), for each prill material under investigation. The plots are gathered in Figure 11. For sample F1, the AN content decreases when moving the grain radially inwards. The closed porosity increases, particularly in the innermost 20 % of the grains, to reach a volume fraction as high as 15%, whilst the average closed porosity content is only 1%. The high-density inclusions are mostly present in the outermost 10 % and innermost 50 %, but are relatively well distributed. All the explosive materials exhibit similar radial distribution profiles for each of their phases. The AN content drops rapidly in the outermost 10 % of the prills, then decreases slowly on the central 10 % to 80 %, and finally drops rapidly over the innermost 20 %. The evolution of the open porosity content is opposite to that of the AN, whilst most of the closed porosity is located in the first 10% of the prills, which corresponds to roughly the outermost 100 µm layer.

## 5. Discussion and Conclusions

As we have seen in the preceding sections and as has been reported in the literature, X-ray computed tomography is a method with great potential and is being increasingly used for non-destructive materials evaluation, in particular for characterising porosity [23,24,25]. However, one of the limitations to a more wide-spread use of XCT is the relative complexity of the data processing. Whilst simple volume fractions or porosity contents can be easily obtained by standard segmentation (although the surface determination is always a critical step [26]), more complex data processing workflows including pre- and post-processing operations complicate the analysis. Another limitation is the difficulty in assessing the robustness, repeatability, and exactitude of the XCT data processing workflows. Here, we have first demonstrated that the filtering employed (pre-processing) helped increase the separation between the peaks in the grey scale histogram (Figure 3c), hence increasing the separation between the materials in the 3D volume. Then, the surface determination strategy based on a watershed segmentation (seed-based) was proven to be repeatable. In fact, upon changing the threshold values used for defining the watershed seeds, we observed a negligible impact on the measured characteristics of the AN prills (details in Table A2 Appendix B). Finally, the assessment of two different specimens coming from the same AN prill batch showed similar results, with values falling within the standard deviations of the respective parameters determined (details in Table A3 Appendix C), demonstrating the overall robustness and repeatability of the data processing workflow proposed here.

**Figure 12:**
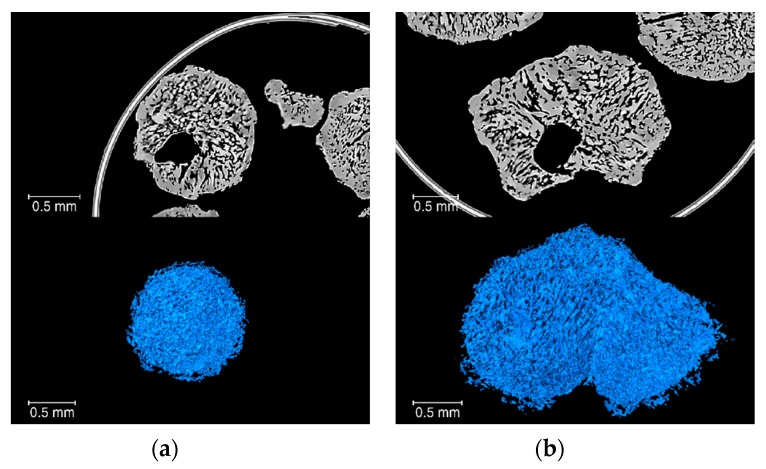
3D renderings: (**a**) open porosity of one prill from specimen E1, with a specific surface area of 139 mm^2^/mm^3^, a shape factor of 6559 and a Euler characteristic χ of −7199; (**b**) open porosity of one prill from specimen E1, with a specific surface area of 139 mm^2^/mm^3^, a shape factor of 25,240 and a Euler characteristic χ of −25,984.

In terms of output, XCT data can provide qualitative information, such as that extracted from the unprocessed 3D reconstructions (Figure 9), where the structure of the prills can be observed. Volume renderings of any of the segmented materials (seen separately, Figure 12) can also provide a good general overview of the complexity of the prill structure in 3D. The qualitative information can be further supported by quantitative information such as volume fraction: fertilizer F1 is the only prill material that has high density inclusions and exhibits very low open porosity (0.02%). On the other hand, the explosive materials have much higher open porosity values, with contents ranging from 20% to 30%. Beyond the structural information, relevant morphological information can be obtained, namely shape factor, Euler characteristic χ and specific surface area. Shape factor values increase and Euler characteristic values decrease with greater open porosity contents, demonstrating that as the open porosity content increases, the whole open porosity network becomes more geometrically complex, i.e., the surface area increases much faster than the volume. This is also quantitatively supported by the specific surface area values: as the oil retention values nearly double between specimen E1 and E3 (8.4% and 14.8%, respectively), there is a doubling of the associated specific surface area values (44.9 mm^2^/mm^3^ and 85.7 mm^2^/mm^3^, respectively). The linear relationship (Figure 10d) reported in this work between the oil retention and the specific surface area determined by XCT, demonstrate that XCT can also be used to predict the performance of AN prills, in addition to giving a full structural and morphological characterisation of the prills. Such structural information can be linked to other relevant parameters, such as explosive sensitivity and velocity of detonation. It was shown in [27] that an optimum porosity content exists, and that above and below that content, velocity of detonation and explosive efficiency are reduced. In this study the optimum porosity was determined to be 30%, related to the total porosity which included both open and closed. In fact, the pore size also plays a role in the detonation capability of the prills [28]. However, in the case of open porosity (the most relevant one), the definition of a “pore size” is still controversial in the literature (see e.g., [11,29]), as it depends on the “pore form” attached to the pore channels, characterizing an open porosity structure. In fact, a typical approach to evaluate “pore sizes” is through the skeletonization of the open porosity structure [30] and then through the calculation of the volume of the spheres with centers in the skeleton and fully filling the channels.

Another important parameter that XCT can deliver is the radial distribution of the different phases. To the best of the authors’ knowledge, this is the first time these types of results are presented. For the explosive materials, it appears that the profiles from E1 through E3 are similar: first a sharp increase in open porosity over the outer 10% and inner 15%–20% of the prill radius, and a much slower increase in-between. In addition, the open porosity content increase between E1 through E3 can be seen as being homogeneously distributed through the radius of the prills, as the open porosity line profiles are globally shifted towards higher volume fractions (between E1, E2 and E3). Finally, another important result for the explosive materials community is the fact that most of the closed porosity in the explosive materials is concentrated within the outermost 100 µm material layer (outer 10% of the prills’ radius. This result could have significant implications, in particular for the sensitivity to explosion and explosion mechanism (hot spot mechanism [9]).

Overall, the results presented here demonstrate that XCT can be successfully applied to the thorough structural and morphological characterisation of AN prills in a non-destructive manner. As XCT can be used to scan virtually any material, it is important to mention that the data processing workflow developed here can be applicable to a broad range of small porous parts and granular porous materials. An advanced data processing workflow was developed, so that both structural and morphological prill parameters could be extracted for each individual prill, whilst performing the segmentation only once for the entire scanned volume. Future work will focus on further investigating qualitative and quantitative morphological data (such as curvature of open porosity (both local and global), local pore/throat size/diameter, and radial evolution of specific surface area), possibly comparing the XCT results to those of conventional techniques, such as BET and mercury porosimetry. This will provide the most relevant metrics for the explosives community to better understand all aspects of the detonation process.

## Supplementary Materials

The following are available online, Video S1: Overview of XCT data processing workflow for ammonium nitrate prills quantitative analysis (uploaded to Zenodo, DOI 10.5281/zenodo.3611339).

## Appendix A

**Table A1:** Overview of the Amira function used in the developed workflow.

Amira Function Name	Operation	Comment
*3D_fill*	Closes holes fully comprised within a selection in 3D	-
*2D_fill*	Closes holes fully comprised within a selection in 2D	For high packing density, over filling can occur.
*Bin_Separate*	Separates individual elements that can be touching each other	Based on a watershed and distance transform
*Border_kill*	Removes the selected elements that are touching a border of the 3D volume	Required so that prills not fully contained within the analysis volume are removed and thus do not skew the values of the measured parameters.
*Label_analysis*	Performs an analysis of the labels to extracts user defined parameters	Parameter of interest are volume, surface area, equivalent diameter, shape factor, and Euler characteristic
*Label_sieve*	Sieves the individual labeled prills based on a discriminating parameter (in this case volume)	Required to remove very small prill fragments
*Labeling*	Gives a unique label to each individual of the binary volume	Required to get a mean value and associated standard deviation
*Distance_transform*	Calculates the distance to the closest boundary from each selected voxel	Required to obtain the radial distribution of prill phases

## Appendix B

An assessment of the sensitivity of the metrics characterised in the present study to changes in threshold values has been performed for sample E1. The exact same data processing workflow was followed apart from the initial label seed definition. For the first test, a “normal” threshold range was selected for each label seed. For the second test, much smaller threshold ranges were selected for each of the label seeds, resulting in more of the voxels remaining to be assigned to a specific label by the watershed algorithm. The results from the two tests and their comparison are presented in Table A2. For the volume fraction values, the relative deviations observed are very small, below 1% of the measured values for AN and open porosity. For the morphological parameters, the relative deviations observed are greater, with values around 10%. However, as the standard deviations of the morphological parameters are generally high (~50%), relative deviations of only 10% of the measured values are deemed satisfactory. These results clearly demonstrate that the watershed segmentation of the filtered XCT data from AN prills is repeatable and that the associated data processing workflow developed here is robust.

**Table A2:** Results of the structural and morphological analysis of sample E1 based on two different segmentation strategies.

Parameter	Segmentation 1	Segmentation 2	Relative Deviation ^1^
Volume fraction AN (%)	77.6 ± 1.1	77.8 ± 1.2	−0.2%
Volume fraction open porosity (%)	22.0 ± 1.2	21.9 ± 1.2	0.8%
Volume fraction closed porosity (%)	0.4 ± 0.1	0.4 ± 0.1	−2.6%
Specific surface area (mm^2^/mm^3^)	44.9 ± 1.9	44.8 ± 2.2	0.2%
AN Euler characteristic (-)	−4589 ± 3327	−4134 ± 3321	9.9%
AN shape factor (-)	1364 ± 811	1241 ± 821	9.0%
Open porosity Euler number (-)	−5807 ± 3688	−5258 ± 3720	9.5%
Open porosity shape factor (-)	10,138 ± 5535	9219 ± 5685	9.1%

^1^ Deviation between Test 1 and test 2, with test 1 taken as reference.

## Appendix C

Three different samples coming from the prill batch E3 were scanned to assess the deviation that can be expected when scanning different samples coming from the same powder. The exact same data processing workflow was followed. Results are shown exemplarily for two scans for the sake of brevity. They all agree within a reasonable interval, which can also be ascribed to genuine materials variability.

**Table A3:** Results of the structural and morphological analysis of sample E3 based on two different samples coming from the same prill batch.

Parameter	Scan 1	Scan 2	Relative Deviation ^1^
Volume fraction AN (%)	67.5 ± 1.9	68.3 ± 2.6	−1.1%
Volume fraction open porosity (%)	32.2 ± 2.0	31.4 ± 2.8	2.5%
Volume fraction closed porosity (%)	0.3 ± 0.1	0.3 ± 0.2	−19.0%
Specific surface area (mm^2^/mm^3^)	85.7 ± 4.2	85.8 ± 3.8	−0.1%
AN Euler characteristic (-)	−29,502 ± 13,257	−37,519 ± 19,229	−27.2%
AN shape factor (-)	5813 ± 2855	6639 ± 3591	−14.2%
Open porosity Euler number (-)	−27,962 ± 12,018	−33,545 ± 15,630	−20.0%
Open porosity shape factor (-)	19,515 ± 9602	23,687 ± 11,076	−21.4%

^1^ Deviation between Test 1 and test 2, with test 1 taken as reference.
